# Para-aortic lymph node metastasis in lower Thoracic Esophageal Squamous Cell Carcinoma after Radical Esophagectomy: a CT-based atlas and its clinical implications for Adjuvant Radiotherapy

**DOI:** 10.7150/jca.51212

**Published:** 2021-01-18

**Authors:** Luxi Ye, Lijun Zhou, Shengping Wang, Lining Sun, Jiazhou Wang, Quan Liu, Xi Yang, Li Chu, Xiaofei Zhang, Weigang Hu, Jie Lin, Zhengfei Zhu

**Affiliations:** 1 Department of Radiation Oncology, Fudan University Shanghai Cancer Center, Shanghai 200032, China.; 2 Department of Oncology, Shanghai Medical College, Fudan University, Shanghai 200032, China.; 3 Department of Radiology, Fudan University Shanghai Cancer Center, Shanghai 200032, China.; 4 Department of Medical Oncology, the Second Affiliated Hospital of Kunming Medical University, Yunnan 650101, China.; 5 Institute of Thoracic Onology, Fudan University, Shanghai 200032, China.

**Keywords:** esophageal squamous cell carcinoma, adjuvant radiotherapy, radiation therapy, lymph node, computed tomography

## Abstract

**Background:** Our previous work showed that para-aortic lymph node (PALN) metastasis was the major failure pattern in lower thoracic esophageal squamous cell carcinoma (LTESCC) patients who presented abdominal LN failure after curative surgery. We thereby aim to generate a computerized tomography (CT)-based documentation of PALNs and to propose a clinical target volume (CTV) for this region.

**Methods:** Sixty-five patients were enrolled. The epicentre of each PALN was drawn onto an axial CT image of a standard patient with reference to the surrounding anatomical landmarks. A CTV for PALN was generated based on the final result of node distribution, and was evaluated for dosimetric performance in three simulated patients.

**Results:** All the studied 248 LNs were below the level of 1.0 cm above the celiac artery (CA), and 94.76% were above the bottom of vertebra L3. Horizontally, 93.33% of the LNs in the celiac level were located within an expansion of 1.5 cm on the CA, and 94.12% of the LNs in the superior mesenteric artery (SMA) level were within 1.5 cm on the left side of the SMA. Below the SMA, all the LNs were behind the left renal vein, left to the right border of the inferior vena cava, and 98.51% of the LNs were medial to the lateral surface of the left psoas major. The proposed CTV could cover 92.74% of the LNs and was dosimetrically feasible.

**Conclusions:** The proposed CTV is the first one to focus on the high-risk area of abdominal failure in LTESCC patients after surgery and can serve as a reference in the adjuvant radiotherapy for LTESCC patients.

## Introduction

Esophageal cancer (EC) is the fourth leading cause of cancer death in China, with more than 90% of them diagnosed as esophageal squamous cell carcinoma (ESCC), leading to different treatment methods compared with the approach used in Western countries. Surgical resection currently remains to be an essential cornerstone in the treatment of ESCC, but surgery alone can result in extremely poor outcomes, with locoregional recurrence being the most frequent failure pattern after definitive lymph node (LN) dissection 1. Postoperative radiotherapy (PORT), despite its controversy, is a common clinical practice in several countries. Studies have shown that PORT, at least in some selected patients, can improve locoregional control and overall survival 2, 3, indicating the necessity for further study.

A reasonable target volume definition is needed for studies that evaluate the value of PORT, and because of which we previously conducted a pooled analysis of failure patterns in patients with thoracic ESCC treated by radical surgery alone 4. The result showed that the three most frequent LN recurrence regions for lower thoracic ESCC (LTESCC) were the cervical and supraclavicular areas, upper mediastinal area, and abdominal para-aortic LNs (PALN) from the level of the celiac artery (CA), which should be recommended for inclusion in the PORT volume for LTESCC patients.

Several studies have reported on the computerised tomography (CT)-based feature of metastatic LNs in the cervical and thoracic region 5, 6, but few have studied the recurrence pattern in the PALN region. In addition, the recurrence pattern in the CROSS study notably showed that preoperative chemoradiotherapy failed to improve celiac and para-aortic region control 7, suggesting that more attention should be paid to the PALNs region. Therefore, based on our previous research, we carry out the current study to propose a CTV suggestion for the PALN region, with reference to the CT-based documentation of PALN metastasis in LTESCC patients after definitive surgery.

## Methods and materials

### Study population

This study was approved by the Institutional Review Board, which allowed us to waive the requirement of written informed consents of individual patients given the retrospective nature of the study. The follow-up abdominal images, including contrast-enhanced CT, magnetic resonance imaging (MRI) and positron emission tomography-CT (PET/CT) of patients with LTESCC who underwent curative surgeries at Fudan University Shanghai Cancer Centre from January 2010 to December 2019 were reviewed. Sixty-five patients were selected on the basis of the following eligibility criteria: (1) having only one primary tumour at first diagnosis and was histopathologically proven to be LTESCC according to the 8th edition of the AJCC/UICC staging system; (2) receiving standard operation as described elsewhere 8; (3) having available follow-up abdominal images which cover the area from the upper border of the liver down to the bifurcation of the abdominal aorta (AA); (4) experiencing PALN metastasis based on the criteria described below; (5) no prior or post-operative radiotherapy to the PALN region; and (6) no other malignancies prior or during the follow-up.

### Diagnostic Criteria of Abdominal LN Metastasis

**Figure 1:**
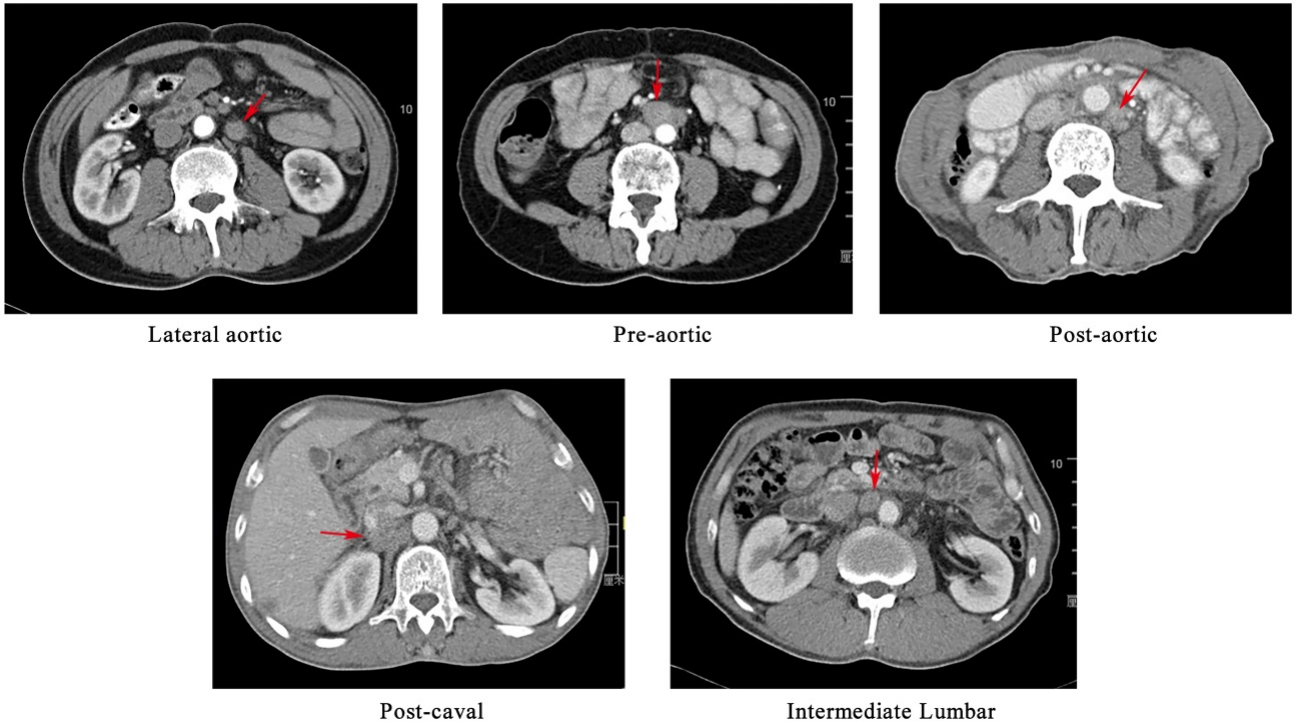
A CT-based illustration of the classification of the metastatic para-aortic lymph nodes.

The preoperative workup, surgical procedure and follow-up of the patients were described elsewhere 8, 9. The suspected metastatic LNs were reviewed by an experienced radiation oncologist and a radiology expert. The positive nodes were identified on the basis of the following features, as described in the previous study^10^, ^11^: (1) round shape with a short axis length of ≥1 cm; (2) presence of an infiltrative margin; (3) continuous increase in number and size compared with those in the previous images; (4) presence of central necrosis or non-homogeneous enhancement; (5) LNs with SUV_max_ value of >2.4 in the PET/CT images; and (6) responsive to anti-cancer treatment. In cases in which patients had more than one follow-up scans, we analysed the first record with positive findings. The PALNs based on the previous study were grouped into left lumbar LNs (including pre-aortic, lateral aortic and post-aortic LNs), intermediate lumbar LNs and right lumber LNs (including precaval, lateral caval and postcaval LNs) 12. The CT-based illustration of the LNs was shown in Figure 1.

### Node mapping

A 59-year-old man who underwent radical esophagectomy for LTESCC with three-field lymphadenectomy was randomly selected as the standard patient. The follow-up abdominal contrast-enhanced CT images, without evidence of abdominal nodal failure, were transferred to the MIM™ software (MIM Software Inc., Cleveland, OH, USA) for the subsequent mapping work. A circle with 5 mm diameter was used to represent the central position of each node. Anatomical landmarks, such as AA, CA, superior mesenteric artery (SMA), inferior vena cava (IVC), vertebral body and left psoas major (LPM), were used as reference. An experienced radiation oncologist and a radiologist worked together to manually draw the LNs onto the corresponding anatomic positions in the axial CT images of the standard patient, with reference to the surrounding anatomical landmarks.

### CTV Generation and Dosimetric Analysis

A proposed CTV for the PALN region was generated based on the final result of the node distribution. The simulation CT scans from three LTESCC patients treated after curative surgery were generated to test the dosimetric feasibility in step-and-shoot intensity-modulated radiotherapy. The planning target volume (PTV) was generated by extending a margin of 1 cm around the CTV based on a previous study about PALN radiation 13. A dose range of 45-50.4 Gy (1.8-2.0 Gy/Fx) is currently recommended by the American National Comprehensive Cancer Network for the PORT settings in ESCC. By referring to the PALN radiation dose used in previous studies 14, 15, a dose of 50.4 Gy in 28 fractions was prescribed in the three simulated cases. The Radiotherapy and Oncology Group guideline was used as the reference for the delineation of organs at risk (OARs) 16, 17. The dose constrains for the OARs are summarised in Supplementary Table S1. The plan optimisation was based on our institutional practice 18.

### Statistics

The data were recorded as categorical or continuous variables and were analysed using IBM SPSS Statistics version 22.0 (SPSS Inc., Chicago, IL, USA).

## Results

### Patient characteristics

The clinical characteristics of the 65 eligible patients are shown in Table 1. All patients received R0 esophagectomy. Forty-two patients (64.62%) received adjuvant therapy, namely, chemotherapy in 26 patients (40%), radiotherapy in 5 patients (7.69%) and chemoradiotherapy in 11 patients (16.92%). Among the patients who received adjuvant radiotherapy, the abdomen region was not part of the CTV in all the patients. PALN metastasis was diagnosed with contrast-enhanced CT in 44 patients (67.69%), contrast-enhanced MRI in 11 patients (16.92%) and PET/CT in 10 patients (15.38%). The median time between the surgery and the record of PALN failure was 15 months (range: 5-79 months).

### Node distribution in relation to anatomical landmarks

**Figure 2:**
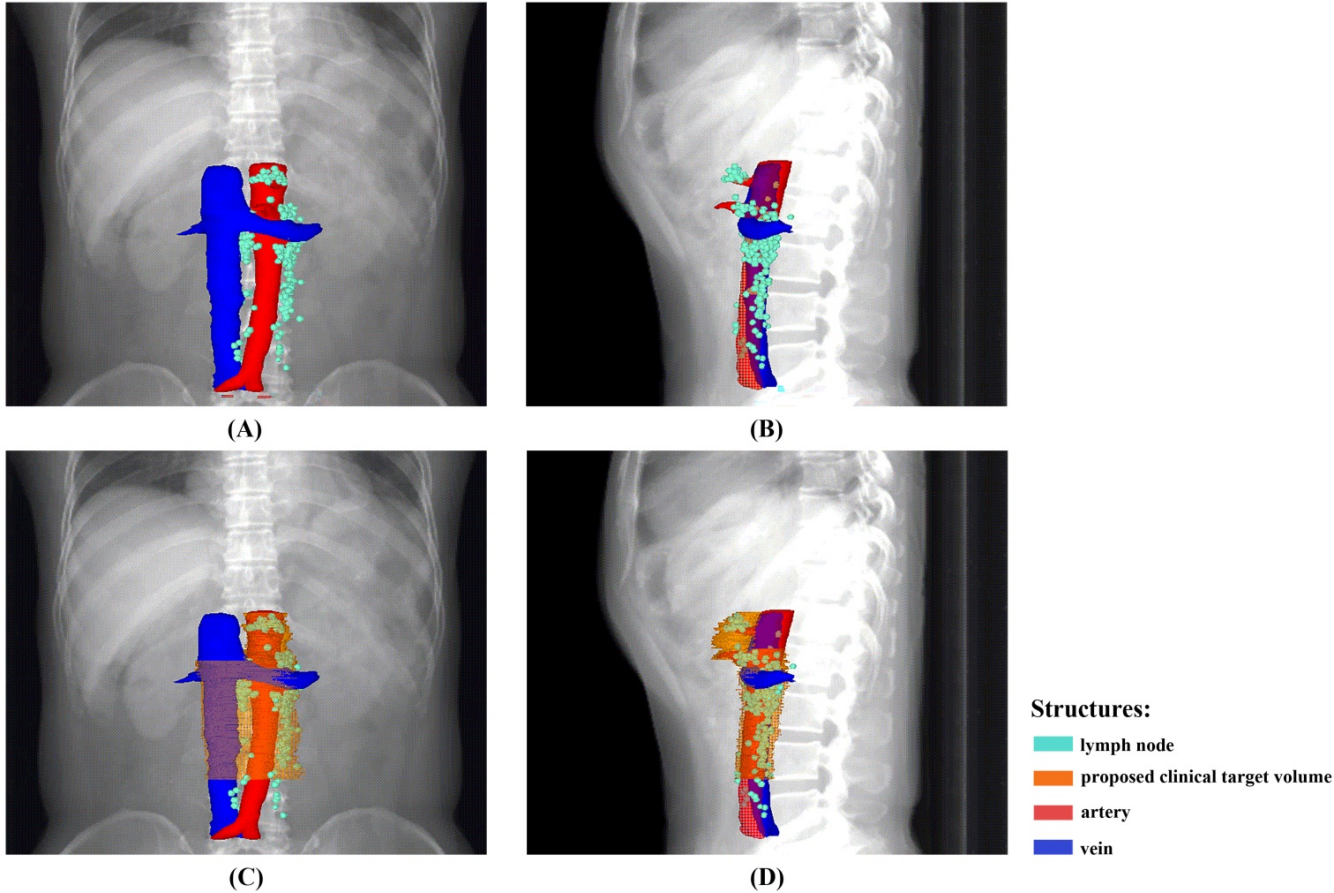
An overview of the recurrence locations in an anterior-posterior (A) and left-right view (B), with an illustration of the node coverage of the proposed CTV in an anterior-posterior (C) and left-right view (D).

**Figure 3:**
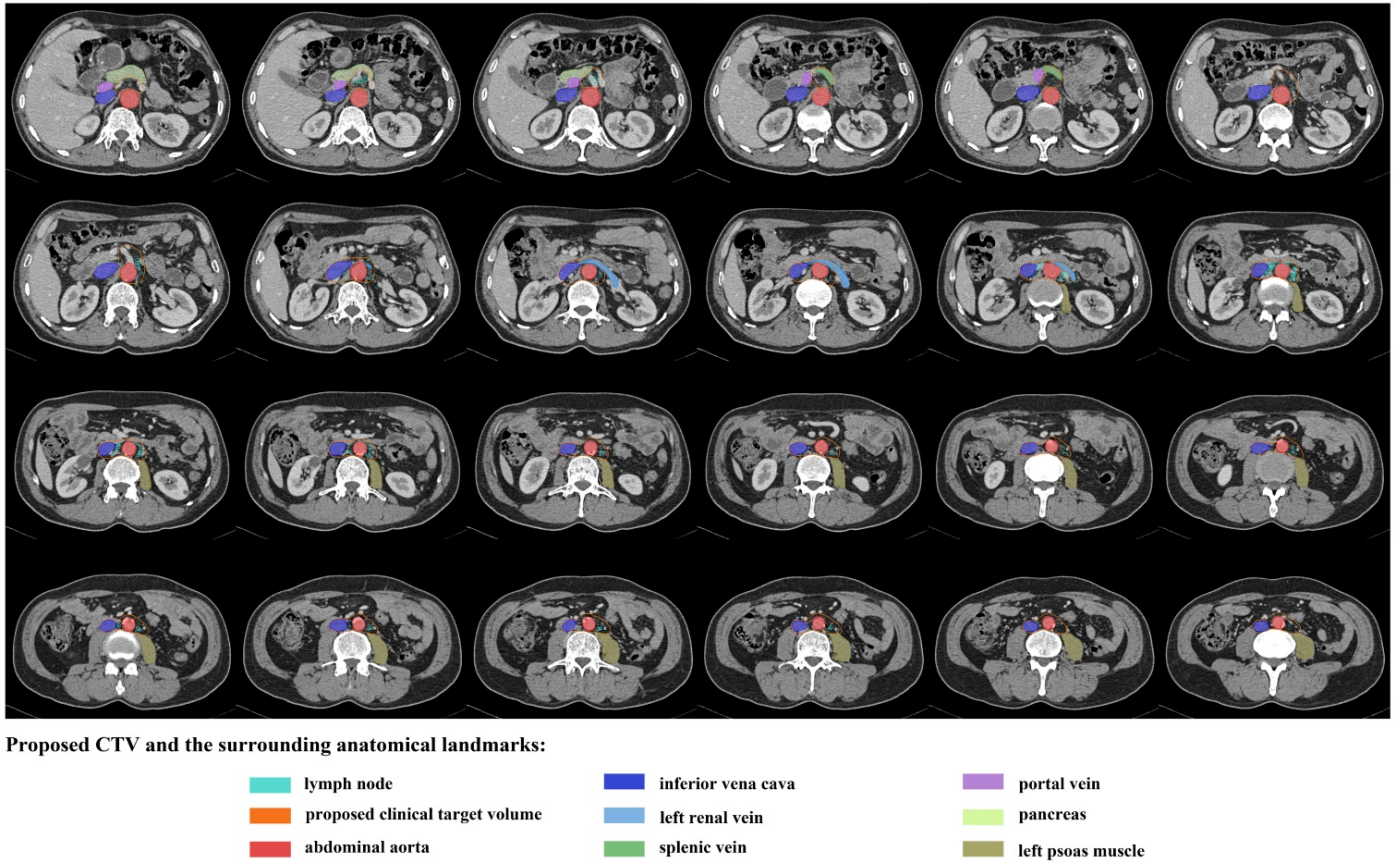
An illustration of proposed CTV contours based on template CT (from standard patient) with 5 mm slice thickness.

A total of 248 nodes were considered malignant. The locations were summarised in Table 2 and shown in Figures 2 and 3. The three most frequent metastasis sites were the lateral aortic region (48.39%), pre-aortic region (22.58%) and intermediate region (19.35%). Sixty-two (95.38%) patients showed left lumbar LNs metastasis (with a median number of 2), and 37 (59.68%) of them had concurrent intermediate or right lumbar LNs metastasis (with a median number of 1). Twenty-eight (43.08%) patients presented PALN metastases with celiac LN failures. The median short axis of the left, intermediate, and right lumbar LNs was 1.25 mm, 0.97 mm, and 1.09 mm, respectively.

In the lateral aortic LNs, the median distance of each LN to the midline of AA was 1.6 cm (range: 0.8-20.8 mm). Amongst the studied patients, six of them showed other abdominal LN metastasis (one patient with pericardial LN, one patient with hepatic communis LN, one patient with peritoneal carcinomatosis, one patient with pericardial LN and retrocrural LN and two patients with hepatic communis LN and retrocrural LN). The LNs outside the PALN region were not depicted in the CT atlas.

To better describe the distribution of the LNs, longitudinally, we divided the area into celiac level (from the celiac trunk to the appearance of SMA), SMA level (from the appearance of SMA to the appearance of the left renal vein) and the region below the SMA (from the appearance of the left renal vein to the bifurcation of the AA). A number of 30, 17 and 201 LNs were seen in the celiac level, SMA level and the region below the SMA, respectively. All the LNs were located below the level of 1.0 cm above the appearance of CA, rendering it an acceptable upper border. As for the caudal edge, 235 (94.76%) LNs were above the bottom of vertebra L3.

Horizontally, in the celiac level, 93.33% of LNs, except two postcaval LNs, were located within an expansion of 1.5 cm on the CA (bounded by the IVC and portal vein on the right, 1.5 cm beyond the left aspect of the AA on the left, the anterior surface of AA posteriorly and the pancreatic body or splenic vein anteriorly). In the SMA level, 94.12% of LNs were within 1.5 cm on the left side of SMA, and all the nodes were behind the anterior edge of the SMA, anterior to the front edges of the vertebral bodies, and left to the left surface of IVC. In the level beneath the SMA, 98.51% of LNs appeared medial to the lateral surface of the LPM, and all the LNs were behind the anterior surface of the left renal vein or adjacent to the anterior surface of the aorta, left to the right border of IVC and anterior to the vertebral or the anterior edge of the LPM. Table 3 summarised the locations of the PALNs in relation to the aforementioned landmarks.

### CTV modification and dosimetric analysis

As shown in Figures 2 and 3, the proposed CTV began at the level 1.0 cm above the CA, then downed to the bottom of vertebra L3. The CTV in the CA level was an area within the 1.5 cm expansion on the CA. Beneath the CA level, the posterior border was the posterior edge of AA in the SMA level and the ventral surface of the vertebral and the front edge of LPM in the region below. The anterior edge was the front end of the SMA in the SMA level and retracted to the anterior surface of the left renal vein, and was set at 0.5 cm anteriorly to the AA below the left renal vein. The right border of the CTV in the SMA level was the connecting line between the anterior of SMA and the left border of IVC, and reached to the right border of the IVC in the region below the SMA. Meanwhile, the left border of the CTV in the SMA level was set at 1.5 cm left to the SMA and to the lateral border of the LPM in the region below. The proposed CTV could cover all of the LNs in 83.08% of the patients and encompassed 92.74% of the studied LNs.

In all the three simulating cases, 95% of the PTV received 99% of the prescribed dose, and all the OARs reached the dosimetric constraints. The mean V45 and V50 of the small intestine was 7.37% and 3.64%, respectively. The average dose of the liver, kidney (bilateral) and small intestines were 10.99 Gy, 10.16 Gy and 20.94 Gy, respectively.

## Discussion

To the best of our knowledge, this study is the first study to focus on the failure pattern of PALN metastasis in LTESCC patients after curative surgery, and it manages to provide a rationale CTV delineating boundary based on cross-sectional CT scans. Only eight abdominal LNs other than the PALNs have been recorded, which is consistent with our previous study 4, suggesting that a CTV focusing on the PALN region is reasonable for selective abdominal LNs radiation in the adjuvant radiotherapy for LTESCC.

Studies have suggested that the PORT for EC patients should be adjusted on the basis of the primary lesion site 19, 20, and selective abdominal LN radiation was also suggested to bring possible benefit to patients with LTESCC 11, 21, 22, but none of those studies have proposed the specific delineation of CTV. In this study, the proposed CTV began at the level 1.0 cm above the CA, then downed to the bottom of vertebra L3, which is similar to the area suggested by Tai et al. 23. Celiac axis LNs, including left gastric, hepatic artery, splenic hilar and celiac artery LNs, were defined as regional LNs in the 7th AJCC staging system, and were suggested to be included in the CTV volume in some studies for LTESCC patients with high risk of recurrence 24, 25. However, various studies have suggested the difference between the celiac artery LNs and other celiac axis LNs. For example, during surgery, LNs in the perigastric area could be easily removed due to the anatomical features, whereas celiac artery LNs were more prone to be omitted due to its difficult accessibility. In fact, despite the surgical dissection, approximately 41.2% of patients reportedly had celiac LN metastasis after R0 radical esophagectomy 22, in contrast to a lower recurrence rate for other upper abdominal LNs 22, 26, 27. Therefore, based on our findings, a 1.5 cm expansion on the CA would be enough to cover the celiac axis LNs region that are at high risk of recurrence.

PALNs below the celiac level were deemed as non-regional LNs for ESCC patients. However, the high rates of metastasis in these region in LTESCC patients after the surgery, as suggested by Wang et al.[10]and Doki et al. 20, should raise our awareness and make us rethink their role in these group of patients. Lower ECs were also reported to have direct drainage routes to PALNs below the celiac level 28, 29, which also explain the relatively large proportion of patients who presented PALN metastasis without celiac LN failure in our study. A similar pattern was also noted by Dorth et al. 30. Besides, PALNs metastasis tended to be sub-clinical. Tanaka reported a 26.7% of PALN metastasis in clinically PALNs (-) patients with lower thoracic esophageal 31. It is worth noting that intraoperative radiotherapy, which included para-aortic area, was reported to significantly improve survival rates in patient with a primary lesion in the lower thoracic or measuring >6 cm in length 32. In our study, 58.46% of patients showed PALN failure without visceral metastasis, indicating the necessity to further stratify LTESCC patients and to identify possible candidate that can benefit from the adjuvant PALNs radiotherapy. What's more, in a recent study investigating chemoradiotherapy with simultaneous integrated boost of radiotherapy dose (SIB-RT) in EC, all patients had nonregional nodal disease, and survival benefit was observed in all the patients receiving SIB-RT, indicating the value of local control in EC patients with nonregional disease 33. PALN metastasis was reported to have a strong correlation with the total number of metastatic lymph nodes, having perigastric lymph node metastases during operations, as well as the depth of tumor invasion 31, 34, 35. It should be noted that these factors were based on the postoperative pathological results, so extrapolation of the results from the current study to preoperative settings should be done carefully. Recent studies have suggested the superiority of involved-field irradiation (IFI, nodal target volume including only the metastatic nodes) compared to elective nodal irradiation (ENI, nodal target volume covering both metastatic lymph nodes and regional nodes) in neoadjuvant treatment for ESCC patients 36, 37. PALN radiation might be more suitable in selected patients with high risk of PALN failure, e.g., patients who did not receive pre-operative radiation and with coeliac node positivity post operatively. Our study can provide the basis for future research to evaluate whether a better control of PALN region could bring survival benefit for LTESCC patients at high risk for PALN failure.

Another issue about the PALNs radiation might be the safety concern, which, however, should not be a problem with the modern radiation therapy equipment. Dosimetric analysis indicated that dose constraint for the small bowel, spinal cord, and other OARs were met with satisfactory. At the same time, experience from cervical cancer also supported its safeness 38, 39. Besides, compared with the available guidelines for distal EC 40, our proposed CTV excludes the retrocrural region due to its uncommon occurrence, as suggested by Pifer et al. 41, which further decreased the dose to the liver and kidney.

Clinically, the application of radiation to PALNs is more often seen in the PORT settings in patients with seminoma 42, pancreatic cancer 17 and cervical cancer 43, 44. Table 4 showed an overview of the anatomical boundaries of the PALN CTVs under different situations. In comparison, a medial border of the left and right iliopsoas muscles was suggested for cervical cancer. However, we assumed that an expansion of the lateral caval region was unnecessary for LTESCC patients, as no LNs would surpass the right margin of the IVC. Our proposed CTV was more similar to the contouring suggestion from pancreatic cancer 45.

The main limitation of this research is its retrospective nature and relatively small volume. Besides, a certain degree of deviation is inevitable due to the manually drawing method. Nowadays, CTV volume for the LTESCC patients after the surgery were decided based on the patient's condition and the physician's judgment. Our study provided suggestions for the selective adjuvant abdomen LNs radiation, but its efficacy when applied with target volume in supraclavicular and mediastinum region need to be further evaluated. Therefore, a larger scale of study is needed to verify the result, in an effort to provide more precise radiation therapy treatments to patients with esophageal cancer.

## Supplementary Material

Supplementary table S1.

## Figures and Tables

**Table 1:** Clinical characteristics of 65 patients

Variables	N
Median age (years)	59
Range	41-75
**Gender**	
Male	58
Female	7
**pT stage^#^**	
T1-2	20
T3-4	45
**pN stage^#^**	
N0-1	34
N2-3	31
**pStage^#^**	
I	5
II	11
III	29
IV	20
**Adjuvant therapy**	
No	23
Chemotherapy	26
Radiotherapy	5
Chemoradiotherapy	11
**Diagnostic radiography**	
contrast-enhanced CT	44
contrast-enhanced MRI	11
PET/CT	10
**Visceral metastasis (Yes/No)**	
No	38
Yes	27

Abbreviations: CT: computed tomography; MRI: magnetic resonance imaging; PET: Positron emission tomography; #Those were the post-operative staging.

**Table 2:** Location of the PALN metastasis

Location	Description	N (%)
**Left Lumbar**		
Pre-aortic	LNs located anterior to the abdominal aorta	56 (22.58%)
Lateral aortic	LNs located to the left of the abdominal aorta	120 (48.39%)
Post-aortic	LNs located posterior to the abdominal aorta	3 (1.21%)
Intermediate Lumbar	LNs located between the IVC and abdominal aorta	48 (19.35%)
**Right Lumbar**		
Pre-caval	LNs located anterior to the IVC	0
Lateral caval	LNs located to the right of the IVC	0
Post-caval	LNs located posterior to the IVC	21 (8.47%)
**Total**		248

Abbreviations: LN: lymph node; IVC: inferior vena cava.

**Table 3:** Location of PALNs in relation of anatomic landmarks

Border	N (%) of LNs out of the border
**Cranial border**	
1.0 cm above the CA	0
**Caudal border**	
2 cm below left renal artery or the bottom of vertebra L2^#^	55 (22.18%)
bottom of vertebra L3	13 (5.24%)
**Anterior border**	
pancreatic body or splenic vein in CA level	0
anterior edge of SMA in SMA level	0
anterior edge of IVC and LRV/5.0 mm anteriorly to the AA	0
**Posterior border**	
anterior edge of AA in CA level	0
posterior edge of AA in SMA level	
anterior edge of vertebral and left psoas major	0
**Lateral border**	
1.5 cm expansion on CA	0
1.5 cm to left border of SMA	1 (5.89%)
lateral border of the left psoas major	2 (1%)
left border of IVC in SMA level	0
right border of IVC beneath SMA level	0

Abbreviations: LN: lymph node; CA: celiac artery; SMA: superior mesenteric artery; AA: abdominal aorta; IVC: inferior vena cava; LRV: left renal vein; #Whichever is more inferior.

**Table 4:** An overview of the anatomical boundaries for the CTV in PALN region among different situations

	PORT for seminoma 42	PORT for pancreatic cancer 17	PORT for cervical cancer 43,44	Our suggested PORT abdominal CTV for LTESCC
Cranial	bottom of vertebral body T10/T11	0.5-1.0 cm expansion of PJ, 1.0-1.5 cm expansion of PV and CA (whichever is most superior)	renal vessels	1.0 cm above the CA
Caudal	inferior border of vertebral body L5	bottom of L2/L3	not specific (most at the level of L3) bifurcation of the aorta	bottom of vertebra L3
Anterior	not specific (AP-PA fields)	1.0-1.5 cm anteriorly to the PV, PJ, CA, and SMA. 2.0-2.5 cm anteriorly to the Aorta.	a 0.75-cm margin in soft tissue around vessels	CA level: splenic vein or pancreatic body; SMA level: anterior edge of SMA; Below the SMA: anterior surface of left renal vein, 0.5 cm anteriorly to the AA
Posterior		anterior border of the vertebral body	anterior border of the vertebral body	CA level: anterior surface of AA; SMA level: posterior edge of AA Below the SMA: ventral surface of the vertebral
Lateral	1.2- to 1.9-cm margin on the aorta and IVC the tips of the transverse processes	0.5-1.0 cm expansion of PJ; 1.0 - 1.5 cm expansion on PV, CA, and SMA; 2.5-3.0 cm to the right, 1.0 cm to the left of the Aorta.	medial border of left and right iliopsoas muscles	CA level: 1.5 cm expansion on the CA; SMA level: 1.5 cm left to the SMA, and right to the connecting line between the anterior of SMA and the left border of IVC Below the SMA: medial to the lateral border of the left psoas major, and left to the right border of IVC

Abbreviations: AA: abdominal aorta; PALN: para-abdominal lymph node; PORT: post-operative radiotherapy; CTV: clinical target volume; IVC: inferior vena cava PJ: pancreaticojejunostomy; PV: portal vein; CA: celiac artery; IVC: inferior vena cava; SMA: superior mesenteric artery.
