# Supplementary material for: Para-aortic lymph node metastasis in lower Thoracic Esophageal Squamous Cell Carcinoma after Radical Esophagectomy: a CT-based atlas and its clinical implications for Adjuvant Radiotherapy
Source: J Cancer. 2021 Jan 18;12(6):1734–41. doi: 10.7150/jca.51212 (PMC7890317; doi:10.7150/jca.51212)
Supplement: Supplementary file 1 — Supplementary table S1. [file jcav12p1734s1.pdf]

Supplementary Table S1. Normal tissue constraints for treatment plans

Structure	Constraints
Spinal cord	maximum dose <45Gy
Kidney	mean dose (bilateral) <18Gy, for patients with two normally functioning kidneys, the dose to 67% of the right kidney and 34% of the left kidney must be <18 Gy; for patients with only one functioning kidney, 15% of the volume of that kidney must receive <18 Gy and 30% must receive <14 Gy.
Liver	V30 <60%<br>mean dose <30Gy
Small intestine	V50<10%, V45<15%
